# The long-term antibody response after SARS-CoV-2 prime-boost vaccination in healthy individuals. The positive influence of extended between-dose intervals and heterologous schedule

**DOI:** 10.3389/fimmu.2023.1141794

**Published:** 2023-04-17

**Authors:** Gretel Naidich, Natalia E. Santucci, Stella Maris Pezzotto, Eduardo A. Ceccarelli, Oscar A. Bottasso, A. Mario Perichón

**Affiliations:** ^1^Centro Unico de Donación, Ablación e Implantación de Organos (CUDAIO), Santa Fe, Argentina; ^2^Instituto de Inmunología Clínica y Experimental de Rosario (IDICER-CONICET-UNR), Rosario, Argentina; ^3^Facultad de Ciencias Médicas, Universidad Nacional de Rosario, Rosario, Argentina; ^4^Concejo de Investigaciones de la Universidad Nacional de Rosario, Rosario, Argentina; ^5^Instituto de Biología Molecular y Celular de Rosario (IBR-CONICET UNR), Rosario, Argentina

**Keywords:** serology, vaccinations, prime-boost, SARS-CoV-2, antibodies

## Abstract

**Introduction:**

Anti-COVID vaccination in Argentina was carried out using different protocols and variations in periods between administrations, as well as combinations of different vaccine platforms. Considering the relevance of the antibody response in viral infections, we analyzed anti-S antibodies in healthy people at different points of time following the Sputnik immunization procedure.

**Methods:**

We attended the vaccination centers in the city of Rosario, which had shorter versus longer intervals between both doses. A total of (1021) adults with no COVID-compatible symptoms (throughout the study period) were grouped according to the gap between both vaccine doses: 21 (Group A, n=528), 30 (Group B, n=147), and 70 days (Group C, n=82), as well as an additional group of individuals with heterologous vaccination (Sputnik/Moderna, separated by a 107-day interval, group D, n=264).

**Results and conclusions:**

While there were no between-group differences in baseline levels of specific antibodies, data collected several weeks after administering the second dose showed that group D had the highest amounts of specific antibodies, followed by values recorded in Groups C, B, and A. The same pattern of group differences was seen when measuring anti-S antibodies at 21 or 180 days after the first and second doses, respectively. Delayed between-dose intervals coexisted with higher antibody titers. This happened even more when using a prime-boost heterologous schedule.

## Introduction

Towards the end of November 2019, a series of pneumonia cases with no precise etiological diagnosis began to be detected in Wuhan, China Shortly after that, it was identified as a severe acute respiratory syndrome coronavirus-2 (SARS-CoV-2) virus. The disease rapidly spread worldwide to the extent that on March 11, 2020, the WHO declared the COVID-19 pandemic (1). The COVID-19 pandemic resulted in a substantial burden on healthcare systems, with specific vaccination being a successful strategy for controlling the spread of the disease. Besides the traditional modalities, like the virus-inactivated or protein/adjuvant approaches, a more novel strategy using viral vectors or nucleic acids was developed (2). Upon 18 months of vaccine implementation, nucleoside-modified messenger RNA (mRNA) vaccines encoding SARS-CoV2 full-length spike and non-replicating adenoviral vector vaccines turned out to be predominantly used in many Western countries.

Currently, replication-deficient adenovirus vectors are being tested as successful vaccine systems (3), as these vectors can deliver the antigen gene without reproduction in the recipient individual (4). This strategy has led to various accessible technological platforms for developing vaccines that generate high titers during vector production and display a high translation efficiency in the host. Adenovirus-based vaccines have already been used for developing experimental systems, clinical trials, and vaccines for general distribution. Systems have been developed against the Zika virus, Ebola, HIV, and influenza (5–8). More recently, different anti-COVID-19 vaccine systems have been produced using the chimpanzee Adenovirus, the human serotype 26, the serotype 5, or a combination of both as prime and boost vaccination protocols (9).

By the end of 2020, Argentina started to administer a two-dose vectored vaccine from the Gamaleya Research Institute (Sputnik V), which employs two different adenovirus vectors, Ad26 and Ad5, expressing the SARS-CoV-2 spike protein. They were initially administered 21 days apart (10).

Evidence from randomized clinical trials or observational studies demonstrated that most anti-COVID-19 vaccines could confer substantial protection against symptomatic and severe diseases with two doses administered 3 to 4 weeks apart (11). Nevertheless, the challenge was vaccine delivery due to supply deficiencies, or limited distribution capacity in some countries (12), and the emergence of more contagious SARS-CoV-2 variants (13). This set the basis for introducing some changes in vaccination policies. They consisted mainly of vaccinating more individuals with the first dose, delaying the second one, or even combining vaccines from different platforms as a sensible approach to improving overall vaccine coverage (14).

In this way, introducing a mRNA or protein vaccine to boost the first dose of an adenovirus vector rendered vaccine programs more responsive to fluctuations. Heterologous prime-boost strategies have been broadly applied to induce protection against several infectious diseases (15).

With regards to the interval between the first and second dose, evidence from clinical trials established that the second dose of the ChAdOx1nCoV-19 vaccine could be given 8–12 weeks after the first dose as it conferred suitable protection levels against COVID-19 infection (16–18).

Considering the relevance of the humoral immune response in viral infections, we aimed to analyze anti-SARS-CoV-2 immunoglobulins titer at different points of time following immunization. For this, we conducted sampling procedures on people who attended the massive vaccination centers designated by the Ministry of Health of the Province of Santa Fe. We conducted these procedures by following the Strategic Plan for Vaccination against COVID-19 in the Argentine Republic. Assuming that the period between the first and second dose was likely to influence the features of the humoral immune response from vaccinated recipients, we studied the levels of anti-S antibodies in individuals with shorter versus longer intervals between both doses.

Given the shortage of the second component of the sputnik vaccine, the Argentine Ministry of Health considered the possibility of implementing a heterologous vaccination using the Moderna vaccine as the second dose. Since this schedule change was voluntary for each potential candidate, we were able to study a group of people vaccinated with Sputnik/Moderna. The response elicited in this group was then compared to those who received two Sputnik doses.

In light that following the vaccine implementation, some participants may have the chance to develop a breakthrough SARS-CoV-2 infection, the humoral immune response to vaccination in people who also experienced a SARS-CoV-2 infection was also investigated.

## Methods

### Patient population

This study is a prospective, longitudinal, observational analysis conducted in a single center. The first two groups (A and B) were health workers from different departments of the Ministry of Health of the Santa Fe Province (primarily administrative staff, doctors, and nurses). Those from Group A were given their second dose 21 days from the first one, as recommended initially. In the case of Group B volunteers, the interval between the first and second dose was extended to 30 days. Group C comprised young adults to whom the second dose was administered 70 days after the first one. An additional group of older adults was also included (Group D). They were subjected to a heterologous prime-boost schedule consisting of Sputnik and Moderna doses, separated by a 107-day interval. The volunteers were given their first vaccine dose between January and July 2021. Participation in the study was voluntary, and all participants signed a written informed consent. The Provincial Bioethics Committee approved the protocol (PROVINCIAL REGISTRY No. 1048). The COVID vaccine regimens and recommendations for the study period in Argentina can be found in the following link: https://bit.ly/3YqnMqY.

### Antibody test

Once the first blood sample was obtained from each volunteer on the day of the first dose, they were scheduled to come back to the CUDAIO Immunogenetics Laboratory 21 days later to collect the second sample. The following sample was also taken at the vaccination facility on the day of administering the second dose. Volunteers had blood drawn 21 days from the second dose and 6 months from the start of the immunization schedule. Serum samples were stored according to our approved protocols (IRB# PRO02737). All samples were identified and registered, and backups of them were kept. Tables containing each individual’s identification and sample tracking numbers were safeguarded. Then, fully anonymous serum samples were obtained by centrifugation, divided into aliquots, frozen, and stored at -80°C until analyzed. All samples were subjected to freezing and thawing before the ELISA analysis. The antibody titer against the receptor binding domain (RBD) of protein S in sera from the blood sample was measured using Immunoassay to quantitatively determine antibodies of the SARS-CoV-2 spike protein using the *Elecsys* Anti-SARS-CoV-2-S Assay (Roche), according to the manufacturer´s instructions. Given that the primary measurement range of the test used is 0.40-250 U/ml, those samples with a detected antibody concentration value > 250 U/ml were automatically diluted 1:10 by the analyzer, for which the maximum measured concentrations were reported as >2500/ml. The samples collected at the moment the first dose was given, 21 days after the first dose at the time the second dose was given, and 21 days after the second dose, were all analyzed simultaneously, whereas the samples obtained at month six were analyzed separately.

### Statistical analysis

Comparisons among groups were performed by non-parametric methods, such as the Kruskal-Wallis analysis, followed by a posthoc test for the multiple comparison approach, when applicable. Paired comparisons were made by the Friedman analysis of variance and the Dunn test. The general linear model was used to evaluate the eventual effects of sex and age on the means of antibody levels for each one of the three antigens. This procedure provides regression analysis and analysis of variance for one dependent variable adjusted by one or more factors. Categorical variables were compared by the chi-square test. Data were considered statistically significant if p< 0.05. Data were analyzed with STATA 6.0 and Prism Graph Pad 4.0 software.

## Results

An outline of the main characteristics of the study groups according to the immunization schedules and blood collection is provided in **Table 1**. Most participants were given two doses of the Sputnik vaccines (groups A, B, and C) except for group D, which received a heterologous prime-boost procedure (Sputnik plus Moderna vaccines).

**Table 1:** A summary of the study groups in terms of vaccination and antibody assessments.

Groups	Timepoint evaluations (the blood sample collection)				
	T1	T2	T3	T4	T5
A	When given the first dose		21 days after the first dose/Coincident with the second dose.	21 days after the second dose (42 days after the first dose)	180 days after the first dose
B	When given the first dose	21 days after the first dose	30 days after the first dose (second dose injection)	21 days after the second dose (51 days after the first one)	180 days after the first dose
C	When given the first dose	21 days after the first dose	70 days after the first dose (second dose injection)	21 days after the second dose (91 days after the first one)	180 days after the first dose
D	When given the first dose	21 days after the first dose	107 days after the first dose (second dose injection)	21 days after the second dose (128 days after the first one)	180 days after the first dose

A: two doses of the Sputnik vaccine separated by a 21-day interval.

B: two doses of the Sputnik vaccine separated by a 30-day interval.

C: two doses of the Sputnik vaccine separated by a 70-day interval.

D: first dose of the Sputnik vaccine, followed by a second Moderna dose separated by a 107-day interval.

The age, gender, and number of people enrolled in the present study are depicted in **Table 2**. While all of them were adult volunteers, group C was composed of younger individuals, followed by participants from groups A and B, whose average ages were around the fifth decade of life. Group D comprised population of adults 10 years older than groups A and B. Despite some differences in sex distribution, all groups showed a clear female predominance (71.4%, B: 71.4%, C: 68.3%, and D: 61.3%)

**Table 2:** Age and gender distribution among the different groups of vaccinated people.

Groups	Sex		Overall Age*
	Males	Females	
**A**	48.18 ± 14.73 (n=151)	44.94 ± 11.68 (n=377)	45.9 ± 12.7 (n=528) *
**B**	51.79 ± 13.41 (n=42)	48.64 ± 13.09 (n=105)	49.54 ± 13.71 (n=147) **
**C**	37.58 ± 2.00 (n=26)	38 ± 2.59 (n=56)	37.87 ± 2.42 (n=82)
**D**	64.79 ± 3.49 (n=102)	64.91 ± 3.32 (n=162)	64.86 ± 3.38 (n=264) ***

Data in years represent means ± SD whereas comparisons were performed by non-parametric tests.

*Statistical differences: A vs B (p<0.05), A vs C, A vs D, B vs C, B vs D, and C vs D: *p<0,05; **p<0,01: ***p<0,001.

**Figure 1:**
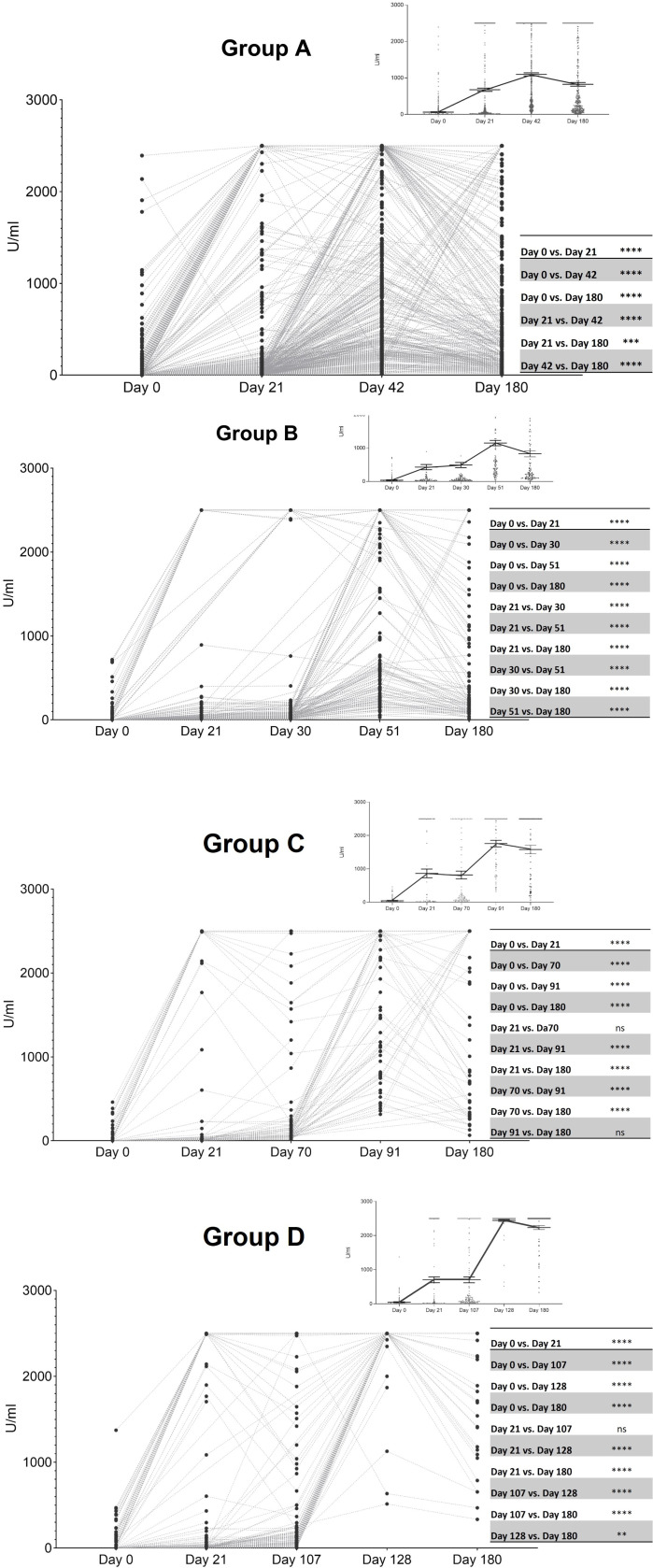
Levels of anti-S antibodies in the four groups of immunized subjects at different time point evaluations. T1: baseline values when administered the first dose; T2: 21 days after the first dose; T3: the time of the second dose; T4: 21 days after the second dose; T5: 180 days after the first dose. Horizontal lines from the top right panel indicate the mean ± standard error of the mean whereas points represent individual data. Grey lines connect paired individual data Friedman test and Dunn´s test for multiple comparison analysis; **, p <.01; ***, p <.001; ****, p <.0001. Group A (two doses of the Sputnik vaccine separated by a 21-day interval): Day 0 (T1) significantly different from the remaining time point evaluations ****, Day 21 (T3) significantly different from Day 42 (T4) (****) and Day 180 (T5) (***), Day 42 (T4) statistically different from Day 180 (T5) (****). Group B (two doses of the Sputnik vaccine separated by a 30-day interval): Day 0 (T1) significantly different from the remaining time point evaluations ****, Day 21 (T2) significantly different from Day 30 (T3) (****) Day 51 (T4) (****), and Day 180 (T5) (****), Day 30 (T3) statistically different from Day 51 (T4) (****) and Day 180 (T5) (****), Day 51 (T4) statistically different from Day 180 (T5) (****). Group C (two doses of the Sputnik vaccine separated by a 70-day interval): Day 0 (T1) significantly different from the remaining time point evaluations ****, Day 21 (T2) significantly different from Day 91 (T4) (****), and Day 180 (T5) (****), Day 70 (T3) statistically different from Day 91 (T4) and Day 180 (T5) (****). Group D (First dose of the Sputnik vaccine, followed by a second dose of Moderna vaccine separated by a 107-day interval): Day 0 (T1) significantly different from the remaining time point evaluations ****, Day 21 (T2) significantly different from Day 128 (T4) (****), and Day 180 (T5) (****), Day 107 (T3) statistically different from Day 128 (T4) (****) and Day 180 (T5) (****). Day 128 (T4) statistically different from Day 180 (T5) (**).

Antibody titer data is presented according to the immunization protocols. Among individuals with a 21-day interval between the first and second doses, the anti-S antibody levels increased from their baseline levels, reaching the highest amounts at T4 (21 days after the second dose). A slight decrease was observed in samples taken 180 days after the first dose (T5) (**Figure 1**, Group A). A similar trend was found when evaluating participants who received the second dose one month after the first one (Group B). In this case, antibody titers also peaked at T4, with data recorded at T5 showing a slight decline in anti-S immunoglobulin values when compared to T4 results (**Figure 1**, Group B). Analysis of the response profile in subjects given two Sputnik V doses, and the most delayed first-second dose interval (Group C), remained in the same direction. It is worth emphasizing that peak titers at T4 and T5 were much higher than those in the two former groups (**Figure 1**, Group C). Further evaluation of data from Group D individuals whose second dose was administered 107 days after the initial one, under a heterologous schedule, showed a similar increase in antibody production at T2 and T3 evaluations. Furthermore, in this group, the maximum concentration of antibodies was also reached in T4, showing the highest antibody levels among the four immunization schedules under analysis. The slight decline in antibody levels at T5 was statistically significant from data recorded at T4 (**Figure 1**, Group D).

**Figure 2:**
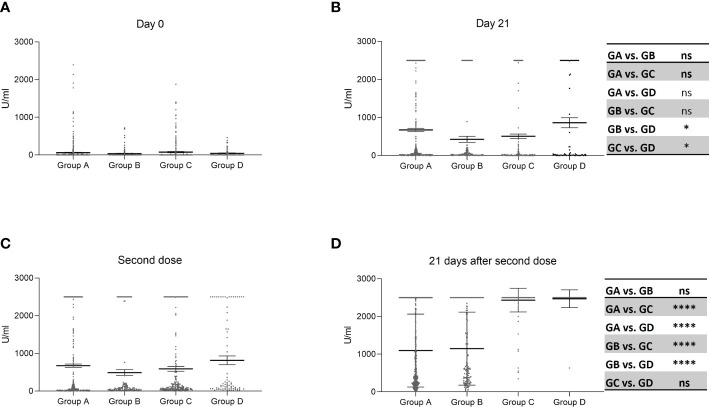
Levels of anti-S antibodies at each time point evaluation according to different immunization protocols. Horizontal lines indicate the mean ± standard error of the mean whereas points represent individual data. Kruskal-Wallis test and posthoc test for multiple comparison analysis; *, p <.05; **, p <.01; ***, p <.001; ****, p <.0001. Group A: two doses of the Sputnik vaccine separated by a 21-day interval Group B: two doses of the Sputnik vaccine separated by a 30-day interval. Group C: two doses of the Sputnik vaccine separated by a 70-day interval. Group D: First dose of the Sputnik vaccine, followed by a second dose of Moderna vaccine separated by a 107-day interval. **(A)**: Day 0 (T1, when administered the first dose) no significant differences. **(B)**: 21 days after the first dose (T2), Group D significantly different from Groups B (*) and C (*). **(C)**: On the day of the second dose (T3) no significant differences.. **(D)**: 21 days after the second dose (T4) Group A significantly different from Groups C and D (****), Group B significantly different from Groups C and D (****).

Next, we compared antibody responses of evaluations at different points in time based on the four protocol immunizations. There were no between-group differences when analyzing baseline levels of specific antibodies (**Figure 2A**). Comparisons among groups B, C, and D, 21 days after the first dose, indicated that the latter group had higher amounts of anti-S immunoglobulins (**Figure 2B**). When analyzing results at the time of administering the second dose, there was a trend of group D to show higher amounts of specific antibodies that did not reach the level of statistical significance (**Figure 2C**). Concerning the 21 days following the second dose of administration, Groups C and D showed much higher concentrations of anti-S immunoglobulins, particularly the second group, than those detected in Groups A and B (**Figure 2D**).

Given that samples were taken from people vaccinated under the current Argentine schedule, we were unable to conform a group of people with second doses administered 21 days after the first ones under a prime-boost heterologous approach. Beyond such limitations, it is worth commenting that we have analyzed a small group subjected to a homologous vaccination protocol using the adenovirus platform. Although the lower number of assessed individuals precluded from a proper statistical analysis, their antibody titers remained in the same range seen in those undergoing the heterologous vaccination schedule (Data not shown).

Data were also analyzed to establish a potential influence of gender and age by applying the general linear model, which provides regression and variance analysis for the antibody titers adjusted by both factors. Data presented in **Table 3** revealed the same pattern of group differences. Nevertheless, in the case of T2 and T3 evaluations, the ages of the participants in the groups also accounted for the statistical differences.

**Table 3:** Antibody levels at each time point evaluation according to the immunization protocols following the adjustment by the general linear model.

Timepoint evaluations	p-value	GROUPS			
		A	B	C	D
T1	0.326	63.0^a^ (43.8 – 87.3)	36.0^a^ (1.5–70.5)	53.1^a^ (3.7 –102.6)	68.2^a^ (31,4 - 104.9)
T2	0.003		314.3^a^ (127.6 – 501.1)	555.0^a^ (240.8 – 869.3)	624.6^a^ (437.8 – 811.3)
T3	0.035	653.3^a^ (565.0 – 741.7)	484.8^a^ (319.1 – 650.4)	747.0^a^ (512.2 – 981.8)	679.4^a^ (490.7 – 868.1)
T4	0.141	1853.1^a^ (1767 – 1939.3)	1906.2^a^ (1758.5 – 2053.8)	2054.9^a^ (1865.9 – 2243.9)	2526.9^a^ 2362.4– 2691.3)
T5	0.642	815.7^a^ (723.1 – 908.2)	826.9^a^ (662.4 – 991.4)	1592.1^a^ (1363.1 - 1821.1)	2301.8^a^ (2111.9 – 2495.7)

a Values indicate means and 95% CI of AU/ml and were sex- and age-adjusted using the general linear model.

A: two doses of the Sputnik vaccine separated by a 21-day interval.

B: two doses of the Sputnik vaccine separated by a 30-day interval.

C: two doses of the Sputnik vaccine separated by a 70-day interval.

D: first dose of the Sputnik vaccine, followed by a second Moderna dose separated by a 107-day interval.

T1: Day 0 (first dose injection); T2: Day 21 from first dose injection; T3: Second dose injection; T4: 21 days after second dose injection; T5: Day 180 after first dose injection.

**Figure 3:**
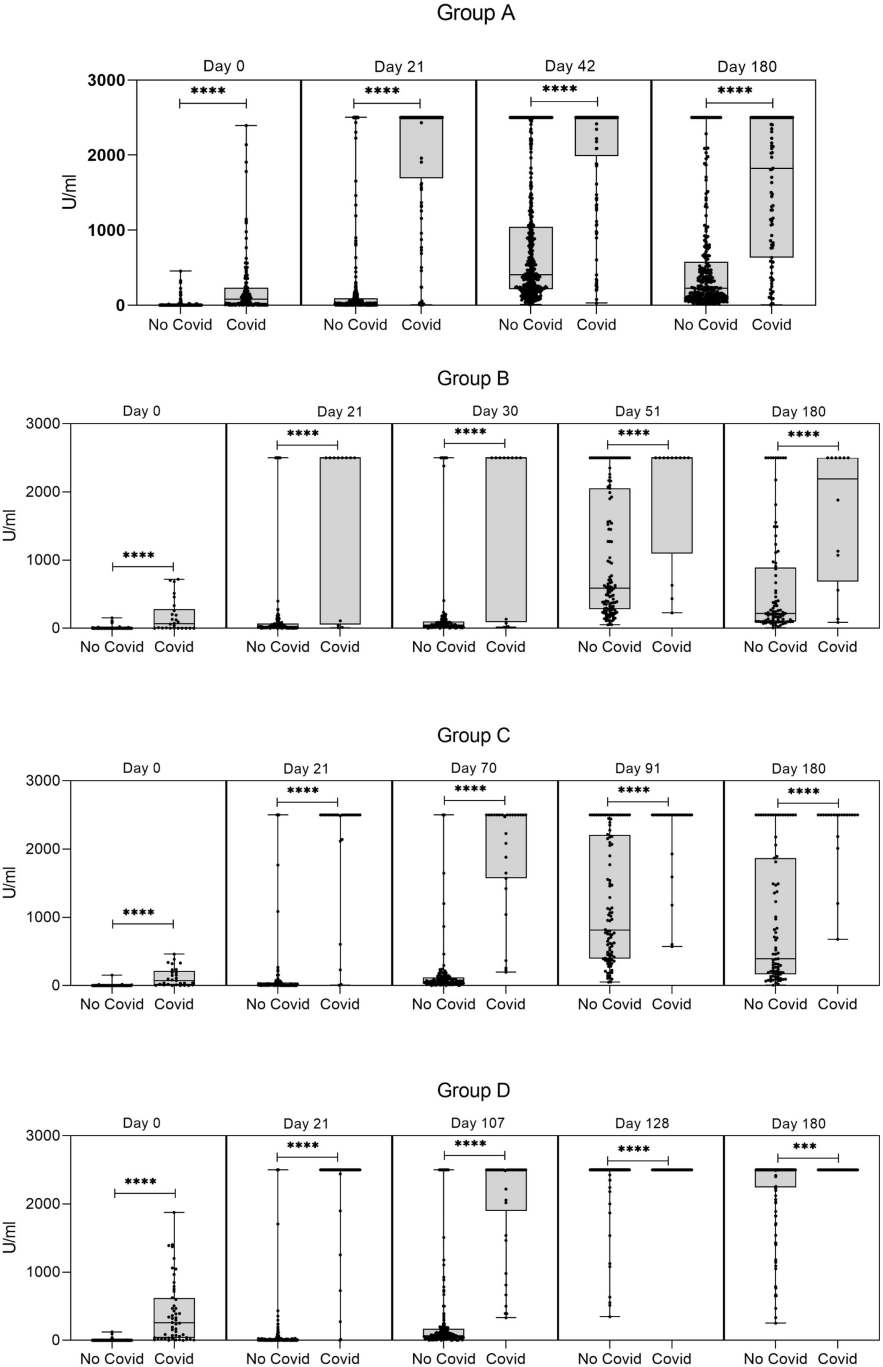
Levels of anti-S antibodies in the four groups of immunized subjects according to whether they experienced a COVID-19 infection, or not. T1: baseline values when administered the first dose; T2: 21 days after the first dose; T3: the time of the second dose; T4: 21 days after the second dose; T5: 180 days after the first dose. Wiskers plots indicate median and interquartile range, with points representing individual data. Mann-Whitney U test; ***, p <.001; ****, p <.0001. Group A (two doses of the Sputnik vaccine separated by a 21-day interval): the Ab titers at T1, T3, T4, and T5 from volunteers with antecedents of COVID-19 infection were significantly different (****) from the ones of uninfected counterparts at all time points evaluations. Days 0, 21, 42, and 180 correspond to T1, T3, T4, and T5, respectively. Group B (two doses of the Sputnik vaccine separated by a 30-day interval: the Ab titers at T1, T2, T3, T4, and T5 from volunteers with antecedents of COVID-19 infection were significantly different (****) from the ones of uninfected counterparts at all time points evaluations. Days 0, 21, 30, 51, and 180 correspond to T1, T2, T3, T4, and T5, respectively. Group C (two doses of the Sputnik vaccine separated by a 70-day interval): the Ab titers at T1, T2, T3, T4, and T5 from volunteers with antecedents of COVID-19 infection were significantly different (****) from the ones of uninfected counterparts at all time points evaluations. Days 0, 21, 70, 91, and 180 correspond to T1, T2, T3, T4, and T5, respectively. Group D (first dose of the Sputnik vaccine, followed by a second dose of Moderna vaccine separated by a 107-day interval). The Ab titers at T1, T2, T3, T4, and T5, from volunteers with antecedents of COVID-19 infection were significantly different from the ones of uninfected counterparts at all time point evaluations (T1, T2, T3, and T4 p<0.0001; T5 p<0.001). Days 0, 21, 107, 128, and 180 correspond to T1, T2, T3, T4, and T5, respectively.

Antibody titers analysis was also done by comparing individuals who experienced COVID-19 infection upon vaccination. As shown in **Figure 3**, these individuals showed higher antibody titers, regardless of the time of sample collection or immunization schedule.

## Discussion

Vaccines against many pathogens, particularly viruses, are thought to mediate protection by generating an antibody response that can neutralize the infecting inoculum and prevent the development of infections (15). Beyond the field efficacy studies, an analysis of the humoral immune response upon specific immunization may also help to understand the immunogenic capability of different vaccine platforms and administration schedules.

Within this framework, our study provides evidence that vaccinated individuals with larger intervals between the first and second doses showed the highest levels of anti-S antibodies in assessments carried out at different time point evaluations following the completion of the prime-boost vaccination. Despite some concern that older individuals may develop a poorer response, it is also worth emphasizing that aged individuals from Group D displayed increased antibody levels. This goes without disregard that age may account for some of the reported differences, particularly at T2 and T3 evaluations where in group B, individuals displayed lower amounts of anti-S antibodies.

Within the context of prime-boost immunization, the first shot which is addressed to elicit an initial immune response to some pathogen proteins is followed by a booster injection to recall immune memory. It may be that a delayed dose (mainly for vaccines using viruses as vectors) would benefit from a possible decline in the levels of anti-vector antibodies, thus decreasing the blocking of the carrier before delivering its information.

Beyond this fact, it is worth emphasizing that antibody concentrations were well preserved regardless of the delay period between the administration of the first and second doses. This lends some support to adenoviral vaccines as a useful immunization approach.

Previous intervention studies employing the ChAdOx1nCoV-19 vaccine revealed increased antibody responses and vaccine efficacy provid the prime-boost interval was extended (16). Flaxman et al. also investigated the immunogenicity persistence in people vaccinated with ChAdOx1 nCoV-19 to find out that the longer the between-doses interval is, the higher the antibody levels are (almost a year vs. 8-12 weeks) (17).

Studies using mRNA vaccines also indicated that longer dose intervals enhanced immunogenicity (19). In the same sense, studies on BNT162b2 mRNA vaccine recipients also demonstrated that an increased interval (6-14 weeks vs. 3-4 weeks) resulted in higher levels of neutralizing antibodies upon administering the second dose (20).

Along with the approach of delayed intervals, heterologous prime-boost COVID-19 vaccination was also promoted to alleviate vaccine supply shortages. Thus, individuals initially primed with an adenovector vaccine further received a second dose, mainly consisting of mRNA vaccines (21). More recently, Goel et al, demonstrated that prolonged intervals between vaccine doses improve the amounts of neutralizing antibodies and memory B cells (22).

Several pieces of evidence indicate that this approach is effective for disease protection (23) and the accompanying immune response. For instance, a longitudinal analysis of the anti-spike immunity found that heterologous vaccinated individuals presented a more robust neutralizing activity irrespective of the SARS-CoV-2 variant (24).

Our study indicates that the same holds true when employing the Sputnik vaccine, which was reported to be effective against COVID-19 (25). To the best of our knowledge, present results provide novel evidence that delayed between-doses intervals of this vaccine coexisted with higher antibody titers, even more when employing a heterologous schedule.

Regarding study limitations, our assessment did not discriminate between non-protective and protective antibodies, whereas findings correspond to a single-center study. We cannot extend our results to what happens in other scenarios regarding antibody titers in vaccinated people. It is also clear that a longer follow-up will help to get a clearer picture of the dynamics of antibody responses, i.e., its durability beyond the classical schedules (26).

The effect of the immunity generated against the vector itself may still be a matter of debate as certain previous seroprevalence studies about adenoviruses may cast some doubts about the usefulness of the vaccine (27, 28). Our results indicate that its impact on the generation of antibodies against the S protein, if present, was negligible since all individuals had easily detectable antibodies using the present vaccination platform.

Finally, we observed that group D had higher antibody titers than groups B and C at day 21, even though all groups received the same first vaccine (**Figure 2B**). While a definite explanation for that is lacking, the particular features of older individuals´ innate and adaptative immune responses may be involved in this regard (29). Among the causes likely to influence the immune response against the administered vaccine, there may be some inability to clear viral particles in the elderly, helping to retain the antigen-carrying vectors for a longer time. An additional and not-mutually exclusive possibility deals with the fact that the elderly display an increased inflammatory response, which may favor the development of the immune response against this viral vector vaccine. We cannot rule out the possibility that these people have an increased response due to a memory generated by earlier exposure to antigens from some coronavirus-related strains, which is more likely to prevail in older people.

Whatever the case, our studies provide a stimulating background to assess whether these findings translate into better protection against COVID-19 disease.

This study was supported by Roche Diagnostics with an unrestricted grant for the contribution of the reagents used to determine the concentration of Anti-SARS-CoV-2 S antibodies.

## Data availability statement

The datasets presented in this study can be found in online repositories. The names of the repository/repositories and accession number(s) can be found below: The datasets generated for this study can be found in the RDA-UNR repository of academic data (Naidich, Gretel; Santucci, Natalia; Pezzotto, Stella Maris; Ceccarelli, Eduardo; Bottasso, Oscar; Perichón, Mario, 2022, “Determinación de inmunoglobulinas totales tras la vacunación anti-SARS-CoV-2 en voluntarios sanos”, https://doi.org/10.57715/UNR/ULPO6K, RDA UNR, V1).

## Ethics statement

The studies involving human participants were reviewed and approved by Comité Provincial de Bioética. Ministerio de Salud de la Provincia de Santa Fe, Argentina. The patients/participants provided their written informed consent to participate in this study.

## Author contributions

GN designed the study, obtained samples and data, and contributed to the writing of the manuscript. NS Analyzed data, wrote and edited the manuscript, and prepare the figures. SP collaborated in the statistical analysis of data. EC contributed to the study design and critically revised the manuscript. OB contributed to the study design, analyzed data and wrote the manuscript. MP designed and supervised the study. All authors contributed to the article and approved the submitted version.
